# Supplementary material for: The Role of Healthcare Providers in Promoting Human Papillomavirus Vaccines among Men Who Have Sex with Men: A Scoping Review
Source: Vaccines (Basel). 2022 Jun 10;10(6):930. doi: 10.3390/vaccines10060930 (PMC9231301; doi:10.3390/vaccines10060930)
Supplement: Supplementary file 1 [file vaccines-10-00930-s001.zip › vaccines-1750192-supplementary.pdf]

vaccines

Table S1. Preferred Reporting Items for Scoping Reviews (PRISMA-ScR) Checklist.

SECTION	ITEM	PRISMA-ScR CHECKLIST ITEM	REPORTED ON PAGE #
**TITLE**			
Title	1	Identify the report as a scoping review.	1
**ABSTRACT**			
Structured summary	2	Provide a structured summary that includes (as applicable): background, objectives, eligibility criteria, sources of evidence, charting methods, results, and conclusions that relate to the review questions and objectives.	1
**INTRODUCTION**			
Rationale	3	Describe the rationale for the review in the context of what is already known. Explain why the review questions/objectives lend themselves to a scoping review approach.	2
Objectives	4	Provide an explicit statement of the questions and objectives being addressed with reference to their key elements (e.g., population or participants, concepts, and context) or other relevant key elements used to conceptualize the review questions and/or objectives.	2
**METHODS**			
Protocol and registration	5	Indicate whether a review protocol exists; state if and where it can be accessed (e.g., a Web address); and if available, provide registration information, including the registration number.	N.A.
Eligibility criteria	6	Specify characteristics of the sources of evidence used as eligibility criteria (e.g., years considered, language, and publication status), and provide a rationale.	2
Information sources*	7	Describe all information sources in the search (e.g., databases with dates of coverage and contact with authors to identify additional sources), as well as the date the most recent search was executed.	2
Search	8	Present the full electronic search strategy for at least 1 database, including any limits used, such that it could be repeated.	2
Selection of sources of evidence†	9	State the process for selecting sources of evidence (i.e., screening and eligibility) included in the scoping review.	2-3
Data charting process‡	10	Describe the methods of charting data from the included sources of evidence (e.g., calibrated forms or forms that have been tested by the team before their use, and whether data charting was done independently or in duplicate) and any processes for obtaining and confirming data from investigators.	3

SECTION	ITEM	PRISMA-ScR CHECKLIST ITEM	REPORTED ON PAGE #
Data items	11	List and define all variables for which data were sought and any assumptions and simplifications made.	3
Critical appraisal of individual sources of evidence§	12	If done, provide a rationale for conducting a critical appraisal of included sources of evidence; describe the methods used and how this information was used in any data synthesis (if appropriate).	N.A.
Synthesis of results	13	Describe the methods of handling and summarizing the data that were charted.	3
**RESULTS**			
Selection of sources of evidence	14	Give numbers of sources of evidence screened, assessed for eligibility, and included in the review, with reasons for exclusions at each stage, ideally using a flow diagram.	3
Characteristics of sources of evidence	15	For each source of evidence, present characteristics for which data were charted and provide the citations.	3
Critical appraisal within sources of evidence	16	If done, present data on critical appraisal of included sources of evidence (see item 12).	N.A.
Results of individual sources of evidence	17	For each included source of evidence, present the relevant data that were charted that relate to the review questions and objectives.	4-14<br>Table 1 & 2
Synthesis of results	18	Summarize and/or present the charting results as they relate to the review questions and objectives.	3-4
**DISCUSSION**			
Summary of evidence	19	Summarize the main results (including an overview of concepts, themes, and types of evidence available), link to the review questions and objectives, and consider the relevance to key groups.	14-16
Limitations	20	Discuss the limitations of the scoping review process.	16
Conclusions	21	Provide a general interpretation of the results with respect to the review questions and objectives, as well as potential implications and/or next steps.	16
**FUNDING**			
Funding	22	Describe sources of funding for the included sources of evidence, as well as sources of funding for the scoping review. Describe the role of the funders of the scoping review.	17

JBI = Joanna Briggs Institute; PRISMA-ScR = Preferred Reporting Items for Systematic reviews and Meta-Analyses extension for Scoping Reviews.

* Where *sources of evidence* (see second footnote) are compiled from, such as bibliographic databases, social media platforms, and Web sites.

† A more inclusive/heterogeneous term used to account for the different types of evidence or data sources (e.g., quantitative and/or qualitative research, expert opinion, and policy documents) that may be eligible in a scoping review as opposed to only studies. This is not to be confused with *information sources* (see first footnote).

‡ The frameworks by Arksey and O’Malley (6) and Levac and colleagues (7) and the JBI guidance (4, 5) refer to the process of data extraction in a scoping review as data charting.
§ The process of systematically examining research evidence to assess its validity, results, and relevance before using it to inform a decision. This term is used for items 12 and 19 instead of "risk of bias" (which is more applicable to systematic reviews of interventions) to include and acknowledge the various sources of evidence that may be used in a scoping review (e.g., quantitative and/or qualitative research, expert opinion, and policy document).

From: Tricco AC, Lillie E, Zarin W, O'Brien KK, Colquhoun H, Levac D, et al. PRISMA Extension for Scoping Reviews (PRISMAScR): Checklist and Explanation. Ann Intern Med. 2018;169:467–473. doi: 10.7326/M18-0850.

Table S2. List of included and excluded items.

**Items included from primary search**		
**No.**	**Authors (years)**	**Title**
1	Colón-López et al. 2012	HPV awareness and willingness to HPV vaccination among high-risk men attending an STI clinic in Puerto Rico
2	Cummings et al. 2015	Catching Up or Missing Out? Human Papillomavirus Vaccine Acceptability Among 18- to 26-Year-old Men Who Have Sex With Men in a US National Sample
3	Kesten et al. 2019	Mixed-methods study in England and Northern Ireland to understand young men who have sex with men's knowledge and attitudes towards human papillomavirus vaccination
4	Meites et al. 2014	HPV vaccine coverage among men who have sex with men - National HIV Behavioral Surveillance System, United States, 2011
5	Merriel et al. 2018	Knowledge and Attitudes of General Practitioners and Sexual Health Care Professionals Regarding Human Papillomavirus Vaccination for Young Men Who Have Sex with Men
6	Moores et al. 2015	Anal cancer screening knowledge, attitudes, and experiences among men who have sex with men in Ottawa, Ontario
7	Nadarzynski et al. 2015	Sexual healthcare professionals' views on HPV vaccination for men in the UK
8	Nadarzynski et al. 2017	UK healthcare professionals' uncertainties, barriers and facilitators to the introduction of targeted human papillomavirus vaccination for men who have sex with men
9	Nadarzynski et al. 2017	Perceptions of HPV and attitudes towards HPV vaccination amongst men who have sex with men: A qualitative analysis
10	Nadarzynski et al. 2018	Men who have sex with men who do not access sexual health clinics nor disclose sexual orientation are unlikely to receive the HPV vaccine in the UK
11	Petit & Epaulard 2020	Men having sex with men and the HPV vaccine in France: A low vaccine coverage that may be due to its infrequent proposal by physicians
12	Rank et al. 2012	Acceptability of human papillomavirus vaccination and sexual experience prior to disclosure to health care providers among men who have sex with men in Vancouver, Canada: Implications for targeted vaccination programs
13	Simatherai et al. 2009	What men who have sex with men think about the human papillomavirus vaccine
14	Stupiansky et al. 2017	Young Men's Disclosure of Same Sex Behaviors to Healthcare Providers and the Impact on Health: Results from a US National Sample of Young Men Who Have Sex with Men
15	Wheldon et al. 2017	HPV vaccine decision-making among young men who have sex with men
16	Wheldon et al. 2018	Physician Communication Practices as a Barrier to Risk-Based HPV Vaccine Uptake Among Men Who Have Sex with Men
17	Wigfall et al. 2018	HPV-related cancer prevention and control programs at community-based HIV/AIDS service organizations: Implications for future engagement

**Items included from reference tracing**		
**No.**	**Authors (years)**	**Title**
1	FitzGerald et al. 2014	The Human Papillomavirus: Men's Attitudes and Beliefs Toward the HPV Vaccination and Condom Use in Cancer Prevention

2	Gerend et al. 2016	Predictors of Human Papillomavirus Vaccination Among Young Men Who Have Sex With Men
3	Gerend et al. 2019	Qualitative Analysis of Young Sexual Minority Men's Perspectives on Human Papillomavirus Vaccination
4	Grace et al. 2019	Economic barriers, evidentiary gaps, and ethical conundrums: a qualitative study of physicians' challenges recommending HPV vaccination to older gay, bisexual, and other men who have sex with men
5	Jaiswal et al. 2020	Gendered Perceptions, and Low Healthcare Provider Communication Around HPV and the HPV Vaccine Among Young Sexual Minority Men in New York City: The P18 Cohort Study
6	Reiter et al. 2010	Acceptability of HPV vaccine among a national sample of gay and bisexual men
7	Reiter et al. 2015	Human Papillomavirus Vaccination Among Young Adult Gay and Bisexual Men in the United States
